# Ultra-Low-Dose Radiotherapy for Palliation of Mycosis Fungoides

**DOI:** 10.1155/2020/4216098

**Published:** 2020-03-24

**Authors:** İpek Pınar Aral, Neşe Göçer Gürok, Aykut Oğuz Konuk, Özlem Üçer

**Affiliations:** ^1^Nevsehir Public Hospital, Radiation Oncology Department, Göreme, Turkey; ^2^Elazığ Fethi Sekin City Hospital, Dermatology Department, Elazig, Turkey; ^3^Elazığ Fethi Sekin City Hospital, Radiation Oncology Department, Elazig, Turkey; ^4^Fırat Univercity, Pathology Department, Elazig, Turkey

## Abstract

*Introduction*. Mycosis fungoides (MF) is a form of primary cutaneous T-cell lymphomas, and radiotherapy (RT) has been used to treat localized/limited lesions of MF. In this case report, the results of low-dose RT applied for palliative purpose are shared. *Case Report*. A 70-year-old male patient was admitted to the outpatient clinic 7 months ago with a generalized itchy rash. The result of the biopsy was reported as mycosis fungoides. Systemic treatment was not performed due to comorbid diseases. The hemibody RT was applied. 2 Gy was given per fraction, with a total dose of 6 Gy. The significant clinical relief was observed with 6 Gy RT. The patient died due to multiorgan failure 2 months later, and no recurrence was observed. *Conclusion*. The palliation was achieved in the advanced MF patient with fractionated 6 Gy hemibody RT for the remaining 2 months of life.

## 1. Introduction

Mycosis fungoides (MF) is the most common form of primary cutaneous T-cell lymphomas [1]. Radiotherapy (RT) has been used to treat localized/limited lesions of MF since 1902. However, large-area skin irradiations using low-energy X-rays or electrons cannot be applied frequently due to equipment, experience, and technical deficiencies [2]. Total skin electron irradiation (TSEI) or total skin irradiation that uses photons (TSI) is performed in a limited number of experienced clinics, especially in MF patients with extensive involvement [3]. TSEI is a method used in the treatment of several dermatological malignant diseases, not only in MF but also in Sézary syndrome or Kaposi's sarcoma; successful results were seen [3–5]. Despite the successful results of RT, the clinician's awareness of this treatment remains low because of concern about side effects [4, 5]. In this case report, the results of ultra-low-dose RT applied for palliative purpose are shared.

## 2. The Case Report

**Figure 1:**
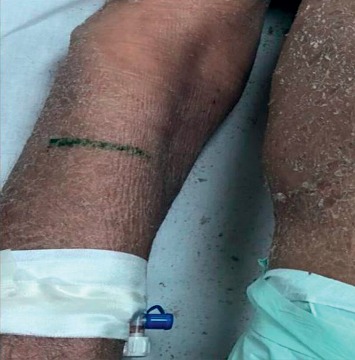
The right upper extremity image of the patient—before RT.

**Figure 2:**
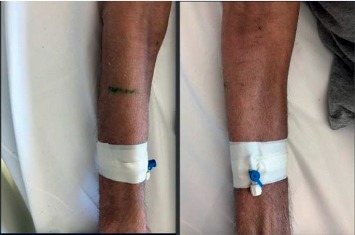
The right upper extremity image of the patient—after RT.

**Figure 3:**
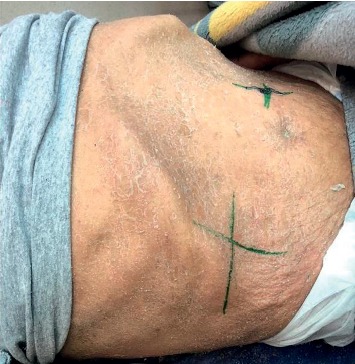
The abdominal image of the patient—before RT.

**Figure 4:**
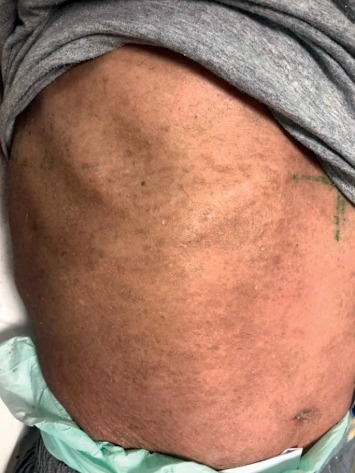
The abdominal image of the patient— after RT.

A 70-year-old male patient was admitted to the outpatient clinic 7 months ago with a generalized itchy rash. A punch biopsy was taken from the thigh region in Firat University Hospital. Epidermis showed significant spongiosis and mild irregular acanthosis. Also, lymphocytes and infrequent neutrophil infiltration were observed in the epidermis. Significant vacuolization was observed in the basal layer. Lymphocytes with irregular hyperchromatic nuclei were detected in the basal layer. Inflammation of mixed cells in the superficial dermis has been reported. Immunohistochemical staining was performed, or CD3, CD4, and CD8 staining was observed. The result of the biopsy was reported as mycosis fungoides. Systemic treatments were not performed due to comorbid diseases (coronary Artery disease (CAD), chronic obstructive pulmonary disease (COPD), paraplegia due to stroke, and hypertension (HT)). The patient also had not started any palliative or symptomatic treatment. He had hypothermia attacks in the last month before admitted to our clinic. On examination, plaques with erythematous, slightly hyperpigmented, widespread flooding were observed throughout the body (Figure 1). The patient was hospitalized in our clinic. The patient was consulted by the dermatology department. The clobetasol propionate, diphenhydramine hydrochloride, and undermine were administered by the dermatologist. This treatment, which was administered before RT, improved the patient's complaints of pruritus and hypothermia. The most complained areas of the patient were bilateral flexor and the upper anterior hemibody. RT was started on the flexor part of the bilateral arms with the most complaints. RT was applied with Elekta Versa HD device, SSD 100 cm, 6 MeV electron energy, 0 gantry angle, using a 20 × 20 applicator. The fraction (frc) dose was 2 Gy, and a total dose of 6 Gy was given. Clinical relief was observed after the first treatment (Figure 2). Following the response, RT was planned for the upper anterior hemibody (Figure 3). Because of the large RT area, right and left dual center radiotherapy was applied. For the right upper anterior hemibody radiotherapy, a 3200 gantry angle of 6 MeV electron energy with an SSD of 100 cm was used. Also, for the left upper hemibody radiotherapy, a 25 × 25 applicator at a 400 gantry angle with the same energy at SSD 100 cm was used. The 2 Gy was given per fraction, and the upper anterior hemibody received a total dose of 6 Gy. Significant clinical relief was observed with 6 Gy radiotherapy and medical treatment recommended by a dermatologist (Figure 4). The patient died due to multiorgan failure 2 months later, and no recurrence was observed in the RT areas.

## 3. Discussion

TSEI and TSI are technically difficult for patients with cutaneous lymphomas. Numerous approaches have been developed to solve “field problems” associated with such a large treatment volume. There is no “ideal” or even standard treatment for extensive skin irradiation. The current recommendations and treatment guidelines suggest several possible options for treatment [4].

Radiotherapy has been used for many years for patients with MF. Radiotherapy is not only effective treatment for the early-stage disease but also contributes to palliative treatment when the disease is advanced. For advanced MF, TSEI is involved in palliation of symptoms such as itching, pain, or dandruff. To date, experiences have shown that the effective dose of RT in the curative treatment of MF is 30 Gy or higher [5, 6]. In the literature, 20–30 Gy/10 frc or 8 Gy/1 frc treatments have been applied for palliative purposes. In advanced MF, the remission of symptoms has been reported in 36–96% of these treatments [6, 7]. Despite the promising results, an important reason why TSEI is not frequently applied in clinical practice is the concern of acute and late side effects [8]. Acute side effects are erythema, edema, hyperpigmentation, and fatigue. Additionally, an increase in the risk of superinfection can be observed [9, 10]. The acute reaction occurs intensely in the second week of treatment. These symptoms are temporary and usually resolve within 2–3 weeks of treatment. Hospitalization may be required according to the prevalence of symptoms [11, 12]. Hyperkeratosis, hyper- and de-pigmentation, and hypohidrosis caused by sweat gland atrophy are late side effects that can be observed due to RT [10]. In our case, no acute side effects were observed.

Historically, total doses of 30 Gy and above were administered. Although the treatment results were successful, severe skin side effects were especially observed. Therefore, low-dose RT started to be tried after 2010 [13–15]. Kroeger et al. achieved adequate treatment results with fewer grade 2 skin toxicities with low-dose (12 Gy) TSEI compared to conventional doses [16]. Additionally, the low total dose is important in terms of the need for a second treatment and improved quality of life [17, 18]. Today, for TSEI, a total dose of 12–36 Gy is often preferred as 4–6 Gy per week, and complete response rates are 90% and above some series [19–21]. After the effectiveness of low dose, phase 2 studies for ultra-low- dose have been tried and ongoing [22].

There is no standard dose and administration schedule of low-dose and ultra-low-dose TSEI for palliative purposes. Since studies on low-dose TSEI are not common in the literature, treatment standards are limited. Besides, there is currently no randomized trial comparing low- and high-dose TSEI. In a study conducted in Copenhagen Dermatology Department, it was reported that 4 Gy low-dose TSEI given in 4 fractions caused partial remission in IB-II MF, but the remission was short-term [9, 23]. In the study of Funk et al., TSEI was applied with doses of 29 Gy and above, and complete remission was observed in 50% of the patients and the remission was permanent for 1–18 months [10]. In our patient, the response was obtained at ultra-low dose (6 Gy), so treatment was terminated, and the patient was followed up closely. In case of recurrence, a second treatment was planned. However, the patient developed cystitis 2 months after the end of treatment and did not improve despite medical treatment. Then, he died of a sudden deterioration of multiple organ failure. No complaints were observed in the RT area during this period. Our case is one of the lowest dose-fractionated palliative wide-field irradiations in the literature.

## 4. Conclusion

In this case evaluation, full palliation was achieved in the advanced MF patient with fractionated 6 Gy hemibody RT for the remaining 2 months of life.
